# Perte de connaissance et épisodes d’épilepsie au Mali. Quel est votre diagnostic?

**DOI:** 10.48327/mtsi.v5i4.2025.765

**Published:** 2025-10-10

**Authors:** Mahamane MARIKO, Mamoudou CAMARA, Kimba B. Abdoul NASSER, Abdoulaye Mody CAMARA

**Affiliations:** 1 Centre hospitalier Luxembourg, Bamako, Mali; 2 Cabinet d’imagerie Médicale, dexploration et de diagnostics (CIMED), Conakry, Guinée; 3 Centre hospitalier universitaire du Mali, Bamako, Mali

**Keywords:** Adolescente, Imagerie cérébrale, Céphalées, Perte de connaissance, Épilepsie, Bamako, Mali, Afrique subsaharienne, Teenager, Brain imaging, Headaches, Loss of consciousness, Epilepsy, Bamako, Mali, Sub-Saharan Africa

## Abstract

Une jeune fille de 15 ans sest présentée au service de neuropédiatrie pour une perte brutale de connaissance avec des antécédents de vomissements, de céphalées intermittentes, de vertiges, de troubles de la vue et de crises d’épilepsie.

Quel est votre diagnostic?

## Abstract (en)

A 15-year-old girl presented to the neuropediatric department with sudden loss of consciousness and a history of vomiting, intermittent headaches, dizziness, visual disturbances, and epileptic seizures.

What is your diagnosis?

## Observation

Une adolescente de 15 ans de confession musulmane s’est présentée en janvier 2025 pour une brutale perte de connaissance au service de neuropédiatrie du Centre hospitalier le Luxembourg à Bamako, (Mali). Première enfant d’une fratrie de trois enfants, sans antécédents pathologiques familiaux particuliers ni voyage effectué hors Bamako, elle résidait à Djikoroni Para, au centre de Bamako.

L’histoire de la maladie remonte à huit ans, marquée par des vomissements épisodiques, des épisodes de crises d’épilepsie, de céphalées frontales avec des vertiges, des flous visuels. Elle prenait de l’ibuprofène 400 mg, du paracétamol 500 mg et de la carbamazépine (posologie non précisée). De multiples consultations à Bamako avaient abouti à plusieurs scanners cérébraux normaux associés à des signes d’épilepsie à l’électro-encéphalogramme (EEG).

À l’admission, la patiente était consciente, apyrétique, avec un indice de masse corporelle de 19,4 (56 kg pour 170 cm), des téguments et des muqueuses bien colorés. L’abdomen était souple, participant à la respiration. La fréquence cardiaque était de 83 par mn et la tension artérielle aux deux bras aux alentours de 110/75 mm Hg. L’échographie cardiaque, l’électrocardiogramme, le fond d’œil et l’EEG étaient normaux. La consultation ophtalmologique n’a pas noté de particularité. La numération formule sanguine ne présentait pas d’anomalie significative. L’examen de la goutte épaisse était négatif pour le paludisme. L’examen parasitologique des selles n’a pas été réalisé faute d’obtention d’échantillon.

## Diagnostic

Ce tableau neurologique progressivement installé depuis plusieurs années doit faire évoquer une atteinte infectieuse ou parasitaire de l’encéphale: granulomes tuberculeux, échinococcose (hydatidose), neurocysticercose, schistosomiase, cénurose.

**Figure 1:**
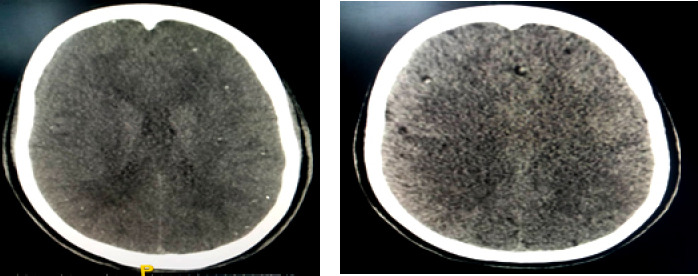
TDM cérébrale en coupe axiale mettant en évidence de muliples nodules calcifiés et kystiques diffus sur le parenchyme cérébral sans rehaussement apres injection de produit de contraste

**Figure 2:**
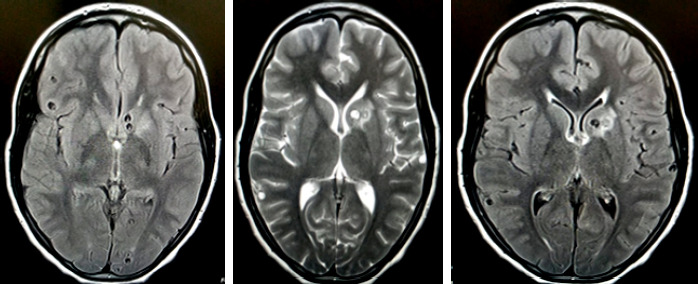
IRM cérébrale mettant en évidence de multiples images kystiques arrondies en hyposignal pondéré T1, hypersignal pondéré T2 et en hyposignal FLAIR de localisation éparse en cortico-sous corticale

Le bilan est poursuivi à l’aide d’un CT-Scan cérébral ainsi qu’une IRM cérébrale. Le scanner cérébral a été réalisé selon le protocole classique: d’abord sans injection, puis avec injection intraveineuse de 84 ml d’Omnipaque (1,5 ml/kg) à 2,5 ml/seconde, puis acquisition au temps artériel à 25 secondes et au temps portal à 70 secondes. Il a permis de mettre en évidence en contraste spontané de multiples microcalcifications nodulaires et kystiques, aux contours nets, de topographie cortico-sous-corticale parenchymateuse cérébrale mesurant 3 mm de grand axe dans la grande majorité des cas. Après injection de contraste, aucun signe d’œdème n’a été noté (Fig. 1). L’imagerie par résonnance magnétique (IRM) en complément de la tomodensitométrie (TDM) avec injection de gadolinium a mis en évidence de multiples images kystiques arrondies en hyposignal pondéré T1, en hypersignal pondéré T2 et en hyposignal FLAIR de topographie éparse en cortico-sous-corticale (Fig. 2).

À la suite de l’imagerie, la sérologie Western Blot réalisée par le laboratoire de biologie médicale Eurofins en France revint fortement positive en faveur d’un téniasis: index IgG = 2,2. L’index IgG est négatif si le l’index IgG est inférieur à 0,8, il est douteux s’il est compris entre 0,8-1,2 et positif lorsqu’il est supérieur à 1,2. La confirmation a été faite par le réactif Cysticercosis Western blot IgG, LDBIO Diagostics avec présence d’au moins 2 bandes parmi les bandes P 6-8, P 12, P 23-26, P 39 et P50-55 kDa.

L’imagerie et le résultat immuno-parasitologique ont conduit à poser le diagnostic de neurocysticercose (NCC).

La patiente a débuté son traitement avec l’albendazole (Albentox) 400 mg par jour pendant 3 jours, puis 3 comprimés par jour une semaine après la première dose associée à de la prednisolone (1 mg/kg) pendant 7 jours et à du praziquantel 50 mg/kg en 2 prises pendant une semaine. Une troisième cure a été effectuée deux mois plus tard. L’évolution clinique à deux mois de traitement a été spectaculaire avec disparition des signes cliniques et une reprise rapide des activités scolaires sans séquelle. Le scanner cérébral de contrôle à trois mois était sans anomalie. La rémission clinique et la normalisation de l’imagerie a été obtenue six mois après le traitement.

## Discussion

La NCC est l’une des parasitoses du système nerveux central les plus répandues dans le monde. Elle peut atteindre 4% de la population générale dans les régions endémiques d’Amérique latine, d’Asie, d’Afrique et d’Europe centrale [6]. En Afrique de l’Ouest, bien qu’une prévalence élevée de cysticercose chez les porcs et chez les humains ait été occasionnellement rapportée, il existe un manque d’approche systématique dans l’étude de la maladie, ce qui peut entraîner une estimation erronée de sa prévalence [6].

L’ingestion de nourriture ou d’eau souillées par des matières fécales contenant des œufs de *Taenia solium* peut provoquer le développement de cysticerques comportant des formes larvaires pouvant migrer vers n’importe quel organe. Dans la plupart des cas, les kystes se développent au niveau des yeux, du système nerveux central, des muscles ou des tissus sous cutanés [7].

Le diagnostic de NCC repose sur des critères absolus ou relatifs:

- démonstration histologique du parasite à partir d’une biopsie d’une lésion cérébrale ou médullaire;
- visualisation des cysticerques sous-rétiniens;
- en neuro-imagerie, plusieurs types d’images sont évocateurs: lésions kystiques sans scolex discernable, lésions avec rehaussement, lésions kystiques multilobées dans l’espace sous-arachnoïdien, calcifications cérébrales parenchymateuses typiques;
- détection d’anticorps anticysticerques spécifiques ou d’antigènes cysticerques par des tests d’immunodiagnostic standardisés.

Un diagnostic probable de NCC peut être posé en l’absence d’examens de neuro-imagerie chez des patients souffrant de crises d’épilepsie et présentant au moins deux critères d’exposition. Notre patiente vivait au Mali, précisément au quartier Djikoroni Para au centre de Bamako (centre urbain).

Il est nécessaire d’une part d’évoquer l’hétérogénéité géographique de cette endémie en lien avec les pratiques alimentaires ainsi qu’avec les pratiques d’élevage et d’hygiène, d’autre part de développer la notion que la religion (et donc les pratiques alimentaires) ainsi que la vie en milieu urbain n’excluent pas la possibilité de cysticercose (séjours en milieu rural dans la famille d’origine, contamination possible *in situ* à Bamako ou dans d’autres centres urbains, etc.). Dans le cas de cette patiente, sa religion a peut-être égaré les premiers traitants dans leur recherche diagnostique. De plus, l’inexpérience des premiers radiologues ayant interprété les scanners cérébraux initiaux n’a pas aidé les cliniciens.

Des rapports indiquent que la région ouest-africaine abrite la plus grande population porcine du continent africain [4], en augmentation de 23% entre 1985 et 2005 [2]. Au Burkina Faso, Carabin *et al.* [1] en 2015 ont trouvé à l’aide du test ELISA 11,5% de cysticercoses chez des villageois et Millogo *et al.* [3] en 2012, ont repéré à l’aide du scanner cérébral 29,4% de cysticercoses chez les sujets épileptiques. Au Nigéria, Weka *et al.* [8] en 2013 ont retrouvé à l’aide du test ELISA (IgG) 9,6% de cysticercoses chez des villageois. Au Sénégal, Secka *et al.* [5] en 2011 ont trouvé à l’aide du scanner cérébral 23,3% de cysticercoses chez des séropositifs HIV.

Enfin, il a fallu trois semaines pour obtenir le résultat du test immunologique, le coût du diagnostic immunologique étant de 25 000 FCFA. En faisant le cumul du coût de tous les examens effectués depuis la consultation outre l’hospitalisation (20 000 FCFA), la goutte épaisse (2 000 FCFA), l’échographie cardiaque (15 000 FCFA), l’électrocardiogramme (5 000 FCFA), le fond d’œil (10 000 FCFA), l’EEG (20 000 FCFA), les examens d’imagerie (CT 45 000 FCFA et IRM 150 000 FCFA) et les médicaments (mébendazole = 2 400 FCFA, prednisolone = 6 200 FCFA la boite, praziquantel 500 FCFA), nous arrivons à une somme de 311 100 FCFA. Cette somme est l’équivalent du salaire mensuel d’un fonctionnaire malien. D’où la nécessité d’étendre les assurances à toutes les couches socio-professionnelles et de créer des fonds de solidarité pour les personnes démunies.

## Conclusion

Ce cas met en relief la nécessité de faire des examens d’imagerie appropriés couplés à l’examen immunologique parasitaire pour parvenir au diagnostic de neurocysticercose. En région d’endémie et également en dehors d’une région endémique, ce diagnostic doit être évoqué par principe devant toute céphalée inexpliquée et réfractaire à tout traitement antalgique, devant des troubles visuels sans anomalie oculaire et en cas d’épilepsie ou d’anomalies de la conscience de survenue brutale.

## Éthique

Le consentement éclairé des parents de la malade pour la réalisation de ce travail a été obtenu de façon verbale.

## Financement

Cette étude n’a bénéficié d’aucun financement.

## Contributions des auteurs

Tous les auteurs ont contribué à l’acquisition, à l’analyse et à l’interprétation des données, ainsi qu’à la rédaction de l’article.

## Liens d’intérêt

Aucun lien d’intérêt lié à ce travail n’a été déclaré.
