# Supplementary material for: Loss of consciousness and epileptic episodes in Mali. What is your diagnosis?
Source: Med Trop Sante Int. 2025 Oct 10;5(4):mtsi.v5i4.2025.765. [Article in French] doi: 10.48327/mtsi.v5i4.2025.765 (PMC12948372; doi:10.48327/mtsi.v5i4.2025.765)
Supplement: Related texts

# Research article: Loss of consciousness and epileptic episodes in Mali. What is your diagnosis?

## Case report

In January 2025, a 15-year-old Muslim girl was admitted to the neuropediatric ward at Centre Hospitalier Le Luxembourg in Bamako, Mali, after experiencing a sudden loss of consciousness. She was the eldest of three children, had no family history of disease, and had not traveled outside Bamako. She lived in Djikoroni Para, in central Bamako.

Her medical history dated back eight years and included vomiting episodes, epileptic seizures, frontal headaches accompanied by dizziness, and blurred vision. She was taking 400 mg of ibuprofen, 500 mg of paracetamol, and carbamazepine (dosage not specified). Multiple consultations in Bamako resulted in normal brain scans and signs of epilepsy on electroencephalogram (EEG). Upon admission, she was conscious and afebrile with a body mass index of 19.4 (56 kg for 170 cm) and well-colored skin and mucous membranes. Her abdomen was soft and participated in respiration. Her heart rate was 83 beats per minute, and her blood pressure in both arms was around 110/75 mmHg. Cardiac ultrasound, electrocardiogram, fundus examination, and EEG results were normal. The ophthalmological consultation revealed no abnormalities. A complete blood count showed no significant abnormalities. The thick smear test was negative for malaria. However, a parasitological examination of the stool could not be performed due to the lack of a sample.

## Diagnosis

This gradually developing neurological condition suggests an infectious or parasitic brain disease, such as tuberculous granulomas, echinococcosis (hydatid disease), neurocysticercosis, schistosomiasis, or crenurosis.

The evaluation continues with a brain CT scan and an MRI. The brain scans were performed according to the standard protocol: first without injection and then with the intravenous injection of 84 mL of Omnipaque (1.5 mL/kg) at 2.5 mL/second. This was followed by acquisitions at the arterial time point at 25 seconds and the portal time point at 70 seconds. The scan revealed spontaneous contrast enhancement of multiple nodular and cystic microcalcifications with clear contours. These microcalcifications were located in the cortical-subcortical parenchymal topography of the brain and measured 3 mm in the long axis in the vast majority of cases. After contrast injection, no signs of edema were noted (Fig. 1). MRI in addition to CT with gadolinium injection revealed multiple rounded cystic images with T1-weighted hyposignal, T2-weighted hypersignal, and FLAIR hyposignal with scattered cortico-subcortical topography (Fig. 2).

After imaging, the Eurofins Medical Biology Laboratory in France performed a Western blot serology test that was strongly positive for taeniasis. The IgG index was 2.2. The IgG index is considered negative if the level is below 0.8, doubtful if the value is between 0.8 and 1.2, and positive if the value is above 1.2. The Cysticercosis Western Blot IgG Reagent (LDBIO Diagnostics) confirmed the results by showing at least two bands among the P6-8, P12, P23-26, P39, and P50-55kDa bands.

**Figure 1:** Axial CT scan of the brain showing multiple calcified and cystic nodules scattered throughout the brain parenchyma without enhancement after contrast injection

**Figure 2:** Brain MRI showing multiple rounded cystic images with low T1 signal intensity, high T2 signal intensity, and low FLAIR signal intensity, scattered throughout the cortical and subcortical regions

The imaging and immunoparasitological results indicated a diagnosis of neurocysticercosis (NCC). The patient began treatment with 400 mg of albendazole (Albentox) per day for three days, followed by three tablets per day one week after the initial dose. This was combined with 1 mg/ kg of prednisolone for seven days and 50 mg/ kg of praziquantel in two doses for one week. A third course of treatment was administered two months later. After two months of treatment, the clinical outcome was spectacular: the patient’s clinical signs disappeared, and they returned to school without sequelae. A follow-up brain scan at three months showed no abnormalities. Clinical remission and normalization of imaging were achieved six months after treatment.

## Discussion

CNS cysticercosis is one of the most widespread parasitic diseases of the central nervous system worldwide. It affects up to 4% of the general population in endemic regions of Latin America, Asia, Africa, and Central Europe [6]. In West Africa, a high prevalence of cysticercosis in pigs and humans has occasionally been reported; however, a lack of systematic study of the disease may lead to inaccurate estimates of its prevalence [6]. Ingesting food or water contaminated with feces containing *Taenia solium* eggs can result in the development of cysticerci containing larval forms that can migrate to any organ. Most often, cysts develop in the eyes, central nervous system, muscles, or subcutaneous tissues [7].

A diagnosis of NCC is based on absolute or relative criteria.

- histological demonstration of the parasite in a brain or spinal cord biopsy;
- visualization of subretinal cysticercus;
- in neuroimaging, the following types of images are suggestive: cystic lesions without discernible scolex, lesions with enhancement, multilobular cystic lesions in the subarachnoid space, typical parenchymal cerebral calcifications;
- detection of specific anticysticercus antibodies or cysticercus antigens by standardized immunodiagnostic tests.

A probable NCC diagnosis can be made in patients with epileptic seizures and at least two exposure criteria in the absence of neuroimaging tests. Our patient lived in Mali, specifically in the Djikoroni Para neighborhood in downtown Bamako.

It is important to note the geographical heterogeneity of this endemic disease in relation to dietary practices, livestock farming, and hygiene practices. It is also important to recognize that religion (and therefore dietary practices) and urban living do not rule out the possibility of cysticercosis (e.g., stays in rural areas with the family of origin, possible contamination in situ in Bamako or other urban centers). In this patient’s case, her religion may have misled the initial doctors in their diagnostic search. Additionally, the inexperience of the radiologists who initially interpreted the brain scans hindered the clinicians.

Reports indicate that West Africa is home to the largest pig population in Africa [4], which increased by 23% between 1985 and 2005 [2]. In Burkina Faso, Carabin *et al.* [1] found 11.5% of villagers to have cysticercosis using the ELISA test in 2015, and Millogo *et al.* [3] found 29.4% of epileptic subjects to have cysticercosis using brain scans in 2012. In Nigeria, Weka *et al.* [8] found that 9.6% of villagers had cysticercosis using the ELISA (IgG) test in 2013. In Senegal, Secka *et al.* [5] found that 23.3% of HIV-positive individuals had cysticercosis based on brain scans in 2011. It took three weeks to obtain the immunological test results, and the immunological diagnosis cost 25,000 CFA francs. The total cost of all the tests performed since the consultation, in addition to hospitalization (20,000 CFA francs), sputum smear (2,000 CFA francs), cardiac ultrasound (15,000 CFA francs), electrocardiogram (5,000 CFA francs), fundus examination (10,000 CFA francs), EEG (20,000 CFA francs), imaging tests (CT scan: 45,000 CFA francs; MRI: 150,000 CFA francs), and medication (mebendazole: 2,400 CFA francs; prednisolone: 6,200 CFA francs per box; praziquantel: 500 CFA francs) is 311,100 CFA francs. This amount is equivalent to the monthly salary of a Malian civil servant. This underscores the importance of extending insurance coverage to all socio-professional groups and creating solidarity funds for the impoverished.

## Conclusion

This case highlights the need for appropriate imaging tests coupled with parasitic immunological testing to diagnose neurocysticercosis. In endemic regions and outside of them, this diagnosis should be considered in cases of unexplained headaches resistant to analgesic treatment, visual disturbances without ocular abnormalities, and cases of epilepsy or sudden abnormalities of consciousness.

## Ethics

The patient’s parents verbally consented to this work.

## Funding

This study did not receive any funding.

## Authors’ Contributions

All authors contributed to the acquisition, analysis, and interpretation of data, as well as to the writing of the article.

## Conflict of interest

The authors declare no conflicts of interest related to this work.
